# Oreo Cookie Treatment Lowers LDL Cholesterol More Than High-Intensity Statin therapy in a Lean Mass Hyper-Responder on a Ketogenic Diet: A Curious Crossover Experiment

**DOI:** 10.3390/metabo14010073

**Published:** 2024-01-22

**Authors:** Nicholas G. Norwitz, William C. Cromwell

**Affiliations:** 1 Harvard Medical School, Boston, MA 02115, USA; 2 Lipoprotein and Metabolic Disorders Institute, Raleigh, NC 27615, USA; wcromwell@velocityclinical.com

**Keywords:** carbohydrates, ketogenic diet, LDL cholesterol, lean mass hyper-responder, lipid energy model

## Abstract

Recent research has identified a unique population of ‘Lean Mass Hyper-Responders’ (LMHR) who exhibit increases in LDL cholesterol (LDL-C) in response to carbohydrate-restricted diets to levels ≥ 200 mg/dL, in association with HDL cholesterol ≥ 80 mg/dL and triglycerides ≤ 70 mg/dL. This triad of markers occurs primarily in lean metabolically healthy subjects, with the magnitude of increase in LDL-C inversely associated with body mass index. The lipid energy model has been proposed as one explanation for LMHR phenotype and posits that there is increased export and subsequent turnover of VLDL to LDL particles to meet systemic energy needs in the setting of hepatic glycogen depletion and low body fat. This single subject crossover experiment aimed to test the hypothesis that adding carbohydrates, in the form of Oreo cookies, to an LMHR subject on a ketogenic diet would reduce LDL-C levels by a similar, or greater, magnitude than high-intensity statin therapy. The study was designed as follows: after a 2-week run-in period on a standardized ketogenic diet, study arm 1 consisted of supplementation with 12 regular Oreo cookies, providing 100 g/d of additional carbohydrates for 16 days. Throughout this arm, ketosis was monitored and maintained at levels similar to the subject’s standard ketogenic diet using supplemental exogenous d-β-hydroxybutyrate supplementation four times daily. Following the discontinuation of Oreo supplementation, the subject maintained a stable ketogenic diet for 3 months and documented a return to baseline weight and hypercholesterolemic status. During study arm 2, the subject received rosuvastatin 20 mg daily for 6 weeks. Lipid panels were drawn water-only fasted and weekly throughout the study. Baseline LDL-C was 384 mg/dL and reduced to 111 mg/dL (71% reduction) after Oreo supplementation. Following the washout period, LDL-C returned to 421 mg/dL, and was reduced to a nadir of 284 mg/dL with 20 mg rosuvastatin therapy (32.5% reduction). In conclusion, in this case study experiment, short-term Oreo supplementation lowered LDL-C more than 6 weeks of high-intensity statin therapy in an LMHR subject on a ketogenic diet. This dramatic metabolic demonstration, consistent with the lipid energy model, should provoke further research and not be seen as health advice.

## 1. Introduction

Carbohydrate-restricted diets, especially ketogenic diets (KD) hold promise for the treatment of obesity-related chronic health conditions, such as type 2 diabetes, as well as for other conditions less related to obesity, including of epilepsy [1], neurodegenerative diseases [2] and mental health conditions [3], polycystic kidney disease [4], and so on. Clinical use cases for KD are rapidly expanding. However, this dietary strategy may cause elevated LDL-cholesterol (LDL-C), a risk factor for atherosclerotic cardiovascular disease (ASCVD) [5]. While the causes of LDL-C rises, when they occur, are likely multifactorial, a particular subgroup of persons adopting KD manifest extreme elevations in LDL-C. The triad of LDL-C ≥ 200 mg/dL, HDL-C ≥ 80 mg/dL, and triglycerides (TG) ≤ 70 mg/dL among individuals on a KD has been termed the lean mass hyper-responder (LMHR) phenotype [6].

It is important to clarify that LMHR is defined solely by the aforementioned lipid triad, and not with a “leanness” criterion. The name derives from an empirical observation that this triad is particularly prevalent among people with a relatively low body mass index (BMI) on a KD. Recent data demonstrate an inverse association between lower BMI and LDL-C elevations in individuals on carbohydrate-restricted diets or KD [6,7].

One explanation for the LMHR phenotype is the lipid energy model (LEM). Briefly, the LEM posits that under conditions of carbohydrate restriction sufficient to deplete hepatic glycogen stores and low body fat, increased levels of circulating fatty acids are resynthesized into TG and exported in very low-density lipoproteins (VLDL) from the liver. Increased numbers of circulating VLDL particles and enhanced lipoprotein lipase (LPL)-mediated catabolism yield increased numbers of LDL particles and LDL-C, increased HDL-C, and decreased TG levels [8].

Consistent with the LEM, prior data suggest that carbohydrate reintroduction is sufficient to reverse the LMHR phenotype and markedly reduce LDL-C levels. Available data show greater LDL-C reductions occur following carbohydrate refeeding among those with lower BMI [6]. This case study crossover experiment tested the hypothesis that carbohydrate supplementation, in the form of Oreo cookies, will lower LDL-C in an LMHR subject to a similar or greater extent than high-intensity statin therapy.

## 2. Materials and Methods

### 2.1. Subject Description

The research subject of this experiment is a 27-year-old male who adopted a KD for the treatment of ulcerative colitis precisely 1520 days before the commencement of this study. Of note, the KD was effective at inducing symptomatic and histologically confirmed colitis remission, consistent with preclinical data [9]. BMI was 20.8 kg/m^2^ at the start of the study. LDL-C levels before starting a KD were 95 mg/dL, increasing to a peak of 545 mg/dL before this experiment when he was particularly lean (lower BMI) and, incidentally, on a low-saturated fat KD. 

Prior full exome sequencing of the subject revealed no pathological variants related to dyslipidemia; prior cholesterol balance testing revealed no evidence of elevated phytosterols or sitosterolemia in the subject’s samples (sitosterol 5.4 mg/L [ref range: <15.0 mg/L)]; and prior coronary artery CT angiogram revealed no evidence of coronary plaque.

The subject is also a medical student, PhD researcher, and the primary author of this manuscript.

### 2.2. Baseline Diet and Lifestyle

The baseline diet was designed to be similar to the subject’s everyday whole foods ketogenic eating pattern and focused on weekly average macronutrient composition and dietary fatty acid profile to provide sufficient flexibility to be maintainable throughout the study arm and within the constraints of the subject’s daily life and duties as a medical student. 80% kCal was derived from fat, with a 1:3 saturated-to-unsaturated fatty acid ratio, 18% kCal from protein, and 2% kCal from carbohydrate (Table 1). Dietary components included seafood, red meat, extra virgin olive oil, dairy, dark chocolate, nuts, and low-carbohydrate vegetables, and food was consumed in roughly two equal meals in an eight-hour daily feeding window between 10:30 and 18:30.

Physical activity was relatively standardized throughout most of the crossover experiment, with 6–7 h of calisthenics and weight training weekly and ~10,000 steps/d. In the final week (week 6) of the statin therapy arm, after LDL-C had plateaued for 3 weeks (weeks 3, 4, and 5), the subject increased his daily step count to ~20,000 steps/d, constituting a sub-experiment testing another prediction of the LEM, i.e. that increasing energy requirements in a KD context with other parameters held constant would increase LDL-C.

### 2.3. Interventions

**Oreo supplementation arm:** Oreo cookies were chosen as a canonical “unhealthy food” and as a demonstration that carbohydrate quality is not as relevant as the simple presence of carbohydrates for reducing LDL-C in the LMHR phenotype. While not pure carbohydrates, Oreos are rich in sugar and contain no other dietary components that would likely reduce LDL-C to any meaningful extent. The dose of 12 Oreo cookies (4 servings; 640 kCal, 28 g fat, 8 g saturated fat, 100 g carbohydrate, 4 g protein) per day split between two meals was chosen to add 100 g/d carbohydrate, with an initial duration of 2 weeks being likely sufficient to see an effect based on the author’s understanding of the LEM and insights from prior self-experimentations and those of other LMHR subjects. 

Throughout the Oreo supplementation arm, exogenous ketones (free d-β-hydroxybutyrate [β-HB], American Ketone) were supplemented to maintain ketosis throughout the study. To eliminate the possibility that circulating ketone bodies were primarily responsible for the subject’s elevated LDL-C, exogenous ketones were dosed at approximately 20 g/d split into four doses and to maintain similar levels to those achieved by the baseline diet via endogenous ketosis (1.2–2.2 mM), except in the latter portion of the night and when morning fasted. Ketone levels were monitored by blood using a Keto–Mojo meter, including one measurement 30–60 min after waking before the first ketone dose as an easily available proxy for repletion of hepatic glycogen stores.

Following the Oreo arm, the subject underwent a 3-month washout period of sustained KD to return to baseline weight and similar cholesterol levels and to ensure no diet carry-over effect.

**Figure 1:**
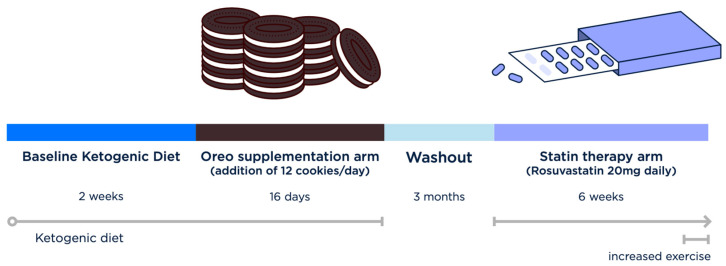
**Crossover experiment timeline.** Following a 2-week run-in period on his habitual ketogenic diet, the subject underwent 16 days of Oreo cookie supplementation, followed by a 3 month washout period and, finally, 6 weeks of 20 mg rosuvastatin daily.

**Statin therapy arm:** Rosuvastatin 20 mg daily was chosen after consultation with the subject’s primary care provider and consultant cardiologist, who recommended a 6-week trial sufficient to give LDL-C levels time to reach a steady state (Figure 1).

### 2.4. Blood Work

Lipid panels were collected at least weekly after a 14 h water-only fast at ~8:30. Given the aim of this experiment was to demonstrate changes in the marker most clinically relevant and understandable to the majority of the medical community and public (LDL-C), only a standard lipid panel was deemed necessary. All the subject’s prior lipid panels over the past 4.5 years on a KD demonstrated a consistent pattern A phenotype with preponderance or large buoyant LDL, small LDL consistently in the optimal-moderate range, and LDL peak size ~230 angstrom (ref range: >222.9 ‘optimal’). Historically, the subject’s LDL-P and ApoB run roughly parallel to his LDL-C, with levels of ~3438 nmol/L and >240 mg/dL, respectively, at his LD-C peak of 545 mg/dL. Other data of interest, taken on his KD before the Oreo intervention, include HbA1c 5.0%, hsCRP < 0.3 mg/L, insulin < 3 uIU/mL, and insulin resistance score ≤ 2 (<33 indicates insulin sensitivity).

As the Oreo arm was designed to be only 2 weeks in duration, and included fewer time points than the statin arm, triplicate labs were collected on sequential days 14, 15, and 16 of the Oreo arm to confirm lipid changes and trends. Consistent Oreo consumption was maintained during that period; thus, the Oreo arm lasted 16 days in total.

### 2.5. Ethics and Safety

The experiment was conducted with the full knowledge and cooperation of the subject’s primary care provider, and with a lipidologist consult (W.C.C.). It was agreed *a priori* that all side effects were to be reported to the subject’s primary care provider and the experiment aborted if his health was in jeopardy.

The Harvard Medical School Institutional Review Board (Protocol: IRB23–1037) provided an exemption for this project on the grounds that n = 1 researcher self-experimentation does not constitute human clinical trials research as defined by the Department of Health and Human Services or U.S. Food and Drug Administration regulations.

## 3. Results

**Figure 2:**
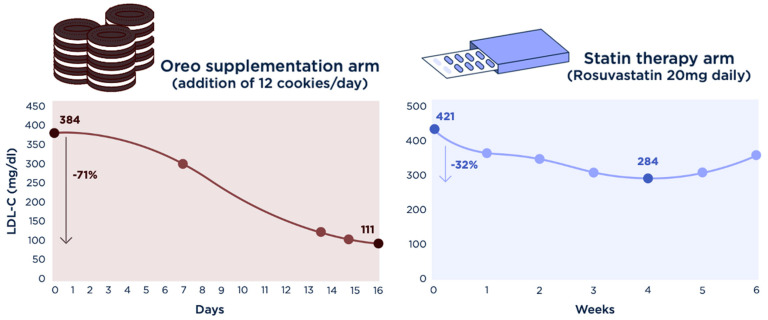
**Effects of Oreo cookies versus Statins on LDL cholesterol (LDL-C)**. 16 days of Oreo cookie supplementation (**Left**) lowered the subject’s LDL-C from 384 to 111 mg/dl (71% reduction). Following a 3-month washout period, the subject underwent 6 months of 20 mg rosuvastatin therapy daily (**Right**), which decreased his LDL-C from 421 to 284 mg/dl at nadir (32.5% reduction). The subject’s LDL-C on a standard mixed diet, prior to adopting a ketogenic diet 4.5 years ago, was 95 mg/dl.

The subject’s baseline BMI was 20.8 kg/m^2^ with LDL-C 384 mg/dL. Oreo supplementation at 12 cookies/d (100 g added carbohydrate) decreased LDL-C to 308 mg/dL during the first week. During this time, β-HB one hour after waking, before the first ketone dose, remained at 0.3–0.7 mM, with an average of 0.5 mM, with a drop to 0.2–0.3 mM in the second week consistent with prolonged and gradual repletion of hepatic glycogen over the first week. After the second week of Oreo supplementation, the subject’s LDL-C dropped to 140 mg/dL (day 14), with repeat tests on subsequent days confirming LDL-C 129 mg/dL (day 15) and 111 mg/dL (day 16). Thus, 16 days of consuming 12 Oreo cookies/d decreased the subject’s LDL-C by 273 mg/dL, a 71% drop, and returned his LDL-C into the ‘normal’ reference range (Figure 2, Table 2), with a continued downtrend towards his pre-KD level of 95 mg/dL. The subject gained 1.5 kg during the Oreo arm. BMI returned to baseline (20.8 kg/m^2^) after *ad libitum* consumption of a KD during the washout period.

Treatment with 20 mg/d rosuvastatin daily ultimately lowered the subject’s LDL-C from 421 mg/dL to 284 mg/dL at 4 weeks, with a plateau in LDL-C to 294, 284, and 295 mg/dL on weeks 3, 4 and 5. There was a moderate increase in LDL-C to 346 mg/dL after the 6th week, during which daily step count was increased from ~10,000 steps/d to ~20,000 steps/d. Thus, peak LDL-C reduction on high-intensity statin therapy was less than half that of the Oreo supplementation, despite 6 weeks of the statin intervention. The only notable side effects of the statin treatment were myalgias with mildly elevated creatine kinase to the mid 300s U/L (risk factors for which, in this case, include lower BMI and physical activity [10]) starting at week 3, through which the subject persevered.

## 4. Discussion

### 4.1. The Lipid Energy Model Predicts Oreo Cookie Treatment Will Lower LDL Cholesterol

The findings of this manuscript are unmistakably curious: Oreo cookie supplementation lowered this subject’s LDL-C by 273 mg/dL, a 71% drop, from baseline. However, this result is predicted by the LEM [8], a mechanistic explanation for the LMHR phenotype. The LEM proposes that under conditions of carbohydrate restriction sufficient to deplete hepatic glycogen stores and relatively low levels of adiposity, free fatty acids are packaged into VLDL particles in the liver and exported as a systemic fuel trafficking system. At peripheral adipocytes and oxidative tissues (skeletal and cardiac muscle), LPL-mediated hydrolysis of TG in VLDL particles produces LDL particles. As the VLDL particles are depleted of TG and become LDL particles, surface components produced by the turnover process are integrated into ApoA acceptor particles [11], i.e., HDL particles, yielding elevated HDL-C. This composite physiology yields a triad of elevated LDL-C, HDL-C, and low TG, which characterizes the LMHR lipid triad.

The LEM is useful in that it makes concrete predictions that can be easily tested, as outlined previously [8]. One prediction is that repleting hepatic glycogen stores should reduce the need for systemic trafficking of TG for fuel, reducing the production of VLDL particles and LDL particles and resulting in lower LDL-C. The model does not emphasize sources or quality of carbohydrates; thus, Oreo cookies should be sufficient to reduce LDL-C in an LMHR subject, as was the hypothesis motivating this case study experiment. The persistence of elevated HDL-C in this subject may have been due to persistent high increased VLDL turnover, despite reduced hepatic VLDL export, stable or increased enteric ApoA production, and differences in the temporal dynamics of particle clearance. The trend toward a decrease in TG to 39 mg/dL (on day 16) is consistent with unpublished data that may result from a relative hyperinsulinemic fasted state and corresponding LPL-mediated release of TG from VLDL into adipocytes in an insulin-sensitive subject. We previously reported a five-person case series of LMHR patients who added carbohydrates to their diet and exhibited an average LDL-C drop of >200 mg/dL, with a maximum drop of 480 mg/dL in the subject with the highest LDL-C on a KD [6]. Thus, the main findings of this experiment are consistent with and extend upon prior data.

While the LEM is a helpful framework for understanding lipid dynamics in LMHR, the cause of the Oreo effect may be multifactorial. For example, Oreo consumption can increase post-prandial insulin and insulin area under the curve. Prior studies show insulin induces LDL receptor pathway activity through multiple mechanisms, including altering interactions between insulin and LDL receptors [12]. However, insulin-induced increase in LDL receptor pathway activity is unlikely to account for the large Oreo effect, which is far greater in relative (−71%) and absolute (273 mg/dL) magnitude than other effects documented outside the LMHR context. Additionally, LDL receptor pathway activity changes would not explain the LMHR triad of markers.

### 4.2. Evidence Is Lacking to Suggest Saturated Fat Is a Driver of the LMHR Phenotype

It is typically assumed that increased saturated fat intake is a major dietary driver of increased LDL-C, when this occurs, in individuals assuming a high-fat KD. However, critical consideration of the available data makes this simple explanation insufficient.

During this experiment, saturated fat levels increased in the study’s Oreo arm; nevertheless, LDL-C levels decreased. Additionally, the subject’s previous peak LDL-C was 545 mg/dL, during which he consumed lower levels of saturated fat (15% of total fat, with 85% of fat intake being monounsaturated or polyunsaturated, i.e., a ~1:5.7 saturated to unsaturated fat ratio). Instead, the higher LDL-C levels previously noted can be explained by corresponding lower body fat, higher activity levels, and greater energy demands consistent with the LEM.

Further consistent with the notion that saturated fat intake is not the main driver of increased LDL-C in lean persons on carbohydrate restricted diets, a recent meta-analysis of 41 randomized trials involving adults consuming < 130 g/d carbohydrates found LDL-C only increased among those studies with BMI < 25 kg/m^2^, whereas those on persons with overweight or obesity class I showed no change in LDL-C, and those in subjects with class II obesity showed a decrease in LDL-C. Furthermore, in a meta-regression accounting for 51.4% of the observed heterogeneity on LCDs, mean baseline BMI had a strong inverse association with LDL-C change (β = −2.5 mg/dL per BMI unit), whereas saturated fat amount was not significantly associated with LDL-C change [7].

Thus, the driver(s) of increased LDL-C in LMHR on KD are much better explained by the LEM than by saturated fat intake.

### 4.3. Increased Physical Activity in Week 6 of Statin Arm Increases LDL-C

Overall physical activity was increased in the final week of statin therapy as a sub-experiment testing another prediction of the LEM: all else being equal, increased lipid energy trafficking demands should increase LDL-C, and HDL-C as a result of LPL-mediated turnover [8]. Before this, the subject’s LDL-C had plateaued to stable levels, with measures on weeks 3, 4, and 5 being within ~4% of each other; therefore, the team felt this would provide useful additional data without confounding the results of the study. Indeed, LDL-C increased by ~50 mg/dL following an increase in daily step count by ~10,000 additional steps/d, consistent with the LEM.

### 4.4. Next Step: A Crossover Trial for LDL Cholesterol Lowering Approaches among LMHR

One advantage of this experiment was the rigor with which it was conducted. The individual subject was capable and motivated to assume a standardized, tracked diet for a long duration, adopt burdensome interventions that may be unethical to impose on recruited volunteers, and rigorously collect data. Thus, this hypothesis-generating experiment is consistent with and expands upon prior data, including our recent meta-analysis of randomized controlled trials, and supports the need for additional research into the LEM and LMHR. This project’s aim was to provoke constructive discussion and inspire a larger-scale crossover trial investigating interventions for LDL-C lowering among LMHR subjects.

### 4.5. Additional Learnings and Limitations

#### 4.5.1. Impact of Oreos on Ketosis

This study anticipated that a 100 g/d dose of Oreos could move the subject out of endogenous ketosis. However, one-hour post-waking fasted β-HB levels persisted at ~0.3 to ~0.7 mmol/L throughout the first week, only dropping out of endogenous nutritional ketosis in the second week of Oreo supplementation. These data suggest that glycogen stores were not sufficiently depleted for at least a week and/or that metabolic adaptations occurred over this timeframe, consistent with prior literature on the extended duration of physiological adaptation to macronutrient shifts [13]. A future trial should consider criteria to ensure a certain degree of glycogen replenishment or sufficient time for adaptation to macronutrient shifts. Options include documenting β-HB ≤ 0.2 mmol/L and/or measuring respiratory quotient (RQ) at intervals to get a sense of the balance of oxidative fuels.

#### 4.5.2. Next Step Study Considerations

The deliberate design choices to add carbohydrates in the form of Oreos, as opposed to a macronutrient switch, deserve commentary. The current experiment serves as a “metabolic demonstration” that a carbohydrate addition can produce findings anticipated by the LEM. Here, an addition was desirable, over an isocaloric macronutrient ‘swap,’ as the latter would necessitate a reduction of protein or, more likely, fat and saturated fat intake. In future studies, intervening with a macronutrient switch may be more practical to enable longer-term compliance among a broader pool of subjects and increase clinical translatability.

### 4.6. Ethical Considerations of Self-Experimentation and the Citizen Scientist Movement

Oreo supplementation should not be considered a beneficial health intervention, and long-term consumption of refined carbohydrates would be very likely to have negative health consequences. This experiment was a “metabolic demonstration.” Accordingly, the data presented in this case study experiment should not imply any form of health advice.

However, while the perception that “playing” with a subject’s cholesterol levels is ethically questionable, the case at hand deserves special consideration. First, the subject and the primary investigator are the same person, and the experiment at hand would have been conducted irrespective of whether the resulting data were peer-reviewed and published. The fact that these results are published merely makes data available for the benefit of other scientists and curious clinicians.

Second, the interventions are generally considered safe, by most standards, at least as acute interventions. Oreo cookies are not a health food, but a foodstuff consumed by the general population routinely and without fanfare. That the subject, a 27-year-old male, ate Oreos is not unusual, unethical, or considered particularly unsafe over 16 days. Conversely, lowering LDL-C via high-intensity statin therapy aligns with standard medical care.

In general, the ethics of self-experimentation and ‘Citizen Science’ (a term referring to a movement among the public towards engaging in self-experimentation, generally in health and fitness) is a topic that deserves further study and debate that is beyond the scope of this manuscript. However, while efforts should be made to ensure public safety, the fact that more people are engaging with their health and personal medical data inquisitively should be encouraged rather than demeaned.

## 5. Conclusions

In this case study experiment on a lean mass hyper-responder on a ketogenic diet, Oreo cookie supplementation lowered LDL-C by 273 mg/dL over 16 days, a 71% drop, as compared to a smaller 137 mg/dL peak reduction, a 32.5% drop, with 6 weeks of high-intensity statin therapy. These findings are consistent with the lipid energy model and highlight the need for further studies on this unique lean mass hyper-responder population.

## Figures and Tables

**Table 1:** Participants daily average dietary macronutrients by study arm. Food was consumed in two roughly equal meals, at ~10:30 and ~18:30, daily. Baseline diet macronutrients equal 80%, 18%, and 2% kCal from fat, protein, and carbohydrate.

	Baseline	Oreo (12 Cookies)	Statin
**Calories**	2616	3350	2616
**Total Fat (g)**	235	263	235
*SAT* (g)	55	63	55
*MUFA* (g)	154	170	154
*PUFA* (g)	25	29	25
**Protein (g)**	118	122	118
**Carb, total (g)**	20	120	20
Fiber (g)	9	11	9

**Table 2:** Lipid changes by study arm. TC, total cholesterol; TG, triglycerides. All units are in mg/dL. * indicates the LDL-C nadir for statin treatment, which was used to represent the statin effect even though it occurred at week 4, not the end of treatment.

	**Baseline**	**Oreo (12 Cookies)**		**Washout**	**Rosuvastatin, 20 mg**						
	**Day 0**	**Day 7**	**Day 16**		**Week 0**	**Week 1**	**Week 2**	**Week 3**	**Week 4**	**Week 5**	**Week 6**
**TC**	494	421	232		539	478	458	406	393	407	468
**LDL-C**	384	308	111		421	354	340	294	284 *	295	346
**HDL-C**	99	102	113		107	116	107	102	102	102	112
**TG**	57	56	39		54	38	54	50	34	49	48
LDL-C reduction, relative (absolute): **−71%** (−273 mg/dL)					LDL-C reduction *, relative (absolute): −32.5% (−137 mg/dL)						

## Data Availability

All research data are published in this manuscript, and the author can be contacted with further specific queries.
